# Comparison of Serum Triglyceride and Cholesterol Levels in Premature Neonates with or without Respiratory Distress Syndrome (RDS)

**DOI:** 10.1155/2021/8893754

**Published:** 2021-02-01

**Authors:** Roya Kelishadi, Behzad Barekatain, Atefeh Fatahi

**Affiliations:** ^1^Department of Pediatrics, School of Medicine, Child Growth and Development Research Center, Research Institute for Primordial Prevention of Non-Communicable Disease, Isfahan University of Medical Sciences, Isfahan, Iran; ^2^Department of Pediatrics, Division of Neonatology, Child Growth and Development Research Center, Isfahan University of Medical Sciences, Isfahan, Iran; ^3^School of Medicine, Isfahan University of Medical Sciences, Isfahan, Iran

## Abstract

**Background:**

Deficiency or reduced transmission of long-chain fatty acids and essential fatty acids may inhibit lung growth and development. We aimed to evaluate and compare serum triglyceride and cholesterol levels in premature neonates with RDS.

**Methods:**

This study is a cross-sectional study performed on premature neonates born in Beheshti Hospital in Isfahan in 2018. Immediately after birth and after umbilical cord clumping, blood samples were taken from the umbilical artery and triglyceride and total cholesterol levels were measured. Those patients with the diagnosis of RDS were transferred to the neonatal intensive care unit (NICU). Data regarding the laboratory results of the lipid profile in patients were compared to that in the other group.

**Results:**

A total number of 100 neonates entered the study and were divided into 2 groups. Analysis of gender and mean gestational ages among the two groups showed no significant differences between the groups (*P* = 0.84 and *P* = 0.28, respectively). Further analysis showed a significant decreased serum cholesterol in the group 1 of patients (*P* = 0.01), but there were no significant differences between the two groups regarding triglyceride levels (*P* = 0.43). There was a significant direct relationship between gestational age and serum triglyceride levels in patients with RDS (*r* = 0.550, *P* < 0.001).

**Conclusion:**

Here, we indicated significantly lower cholesterol levels in the cord serum of premature neonates with RDS compared to non-RDS premature neonates. Our data also showed a significant direct relationship between gestational age and serum triglyceride levels in patients with RDS. These data were in line with the previous studies.

## 1. Introduction

Respiratory distress syndrome (RDS) is one of the most common respiratory problems in infants, and its prevalence rate worldwide accounts for about one percent of all deliveries [1, 2]. According to epidemiologic studies in the United States, 80-60 thousand babies are born with RDS every year, of which about 6-12 thousand die [3, 4]. Based on evidence, RDS or its complications are a major cause of death in the first month of life and accounts for about 30% of all neonatal deaths [4]. About 20 percent of surviving infants with RDS develop chronic lung disease in the later ages, and studies have indicated an inverse relationship between gestational age, birth weight, and risk of RDS [5, 6]. Respiratory distress syndrome occurs in infants who lack the production and secretion of surfactant in the lungs. Surfactant prevents the lower airways from closing after the delivery [7]. Clinical manifestations of RDS are mostly due to the reactions to decreased lung activity. Infants with RDS may have a combination of several clinical symptoms, including grants, tachypnea, hypotension, intercostal retraction, pulmonary edema, decreased respiratory sounds, central cyanosis, and decreased urination within 24-48 hours after birth [8, 9]. As the disease progresses and the clinical condition worsens, the granting decreases due to increased respiratory work and fatigue. Irregular breathing and apnea attacks usually indicate respiratory fatigue and require prompt intervention [10].

In recent decades, new therapies have been developed in infants with RDS, including the protocol for administering steroids to the mother to accelerate fetal lung maturation, the administration of surfactants to premature infants with RDS, and also mechanical ventilation [11]. Lipid metabolism plays an important role in fetal development in the late stages of pregnancy, which includes the growth and increase of adipose tissue in the intrauterine life of the fetus [12]. Lipid metabolism also plays a significant role in the transport of cholesterol to the adrenal glands of the fetus to produce hormones. Studies have indicated that the elevated amniotic fluid lecithin levels are associated with the maturation of respiratory function and changes in phospholipid levels in the amniotic fluid [13].

Deficiency or reduced transmission of long-chain fatty acids and essential fatty acids may inhibit fetal growth and development, one of the effects of which may be on fetal lung development, leading to RDS after birth. Serum lipid levels are evidence of decreased essential fatty acids and long-chain unsaturated fatty acids that can inhibit fetal growth in the uterus and delay fetal lung maturation [14]. Studies have shown that amniotic fluid cholesterol levels are lower in mothers whose children have RDS than in other mothers and suggest that amniotic fluid cholesterol levels in mothers may be a predictor of RDS in infants [15]. Studies have shown that high levels of High Density Lipoprotein (HDL) and Low Density Lipoprotein (LDL) stimulate the production of type II cells to secrete phosphatidylcholine, which is the major phospholipid compound in surfactant needed for fetal lung maturation [16, 17]. Researchers also believe that RDS is generally associated with changes in lipid levels, and measuring its level can assess the risk of developing RDS in infants [18].

Due to the prevalence of respiratory distress syndrome in infants, especially in preterm infants, and the hypotheses that lipid concentrations are lower in preterm infants with RDS compared to other infants, and also due to different results in different studies, we assume that by measuring these factors in preterm infants, we could be able to estimate the risk of developing RDS. This issue is critical in preventing the occurrence of RDS by modifying the level of these factors.

## 2. Methods and Material

This study is a descriptive-analytical cross-sectional study performed on premature neonates born in Beheshti Hospital of Isfahan University of Medical Sciences in Isfahan in 2018-2019.

Our inclusion criteria were premature neonates with 26 to 34 weeks gestation age, no asphyxia at birth, Apgar score more than 7 in 5 minutes of birth, delivery by cesarean section, and filling up the written informed consent by the parent to participate in the study. The exclusion criteria were having major congenital anomalies, inability to send laboratory tests, and identifying another cause for respiratory symptoms other than RDS during the course of the disease, such as pneumonia.

Neonates were entered based on inclusion and exclusion criteria. Before the study begins, all parents signed a written informed consent, and the study processes were explained in detail to parents. Immediately after birth and after umbilical cord clumping, blood samples were taken from the umbilical artery and were delivered to the laboratory in order to measure the lipid profiles of all premature patients. Triglyceride and total cholesterol were measured in all patients using standard laboratory methods. It should also be noted that all measurements were performed using the same automatic analyzer.

Those patients with the diagnosis of RDS were assigned to group 1 and were transferred to the neonatal intensive care unit (NICU). The diagnosis of RDS was made by an expert pediatrician using clinical findings and chest X-ray imaging. Those patients who also had inclusion criteria but did not have RDS were assigned to the control group. Patient recruitment continued until the appropriate study population (50 patients in each group) was reached. We should also note that data of all patients were also collected after patients entered the study. These data included gestational age, weight and height by the time of birth, sex, any maternal medical issues by the time of pregnancy, drug history of mothers during pregnancy, the main reason of patient's admission in NICU, Apgar score of neonates, and type of delivery. Data regarding the laboratory results of lipid profile in patients were also noted. Data were analyzed using SPSS software version 24.

## 3. Results

A total number of 100 neonates entered the study and were divided into 2 groups of neonates with RDS (group 1) (50 patients) and neonates without RDS (group 2) (50 patients). Group 1 consisted of 27 boys (54%) and 23 girls (46%), and group 2 consisted of 28 boys (56%) and 22 girls (44%). Analysis of gender among the two groups showed no significant differences between groups (*P* = 0.84 using chi-square). Mean gestational ages were 30.4 ± 2.08 weeks and 30.8 ± 2.09 weeks in groups 1 and 2, respectively, but there were no significant differences (*P* = 0.28 using independent *t*-test).

**Figure 1:**
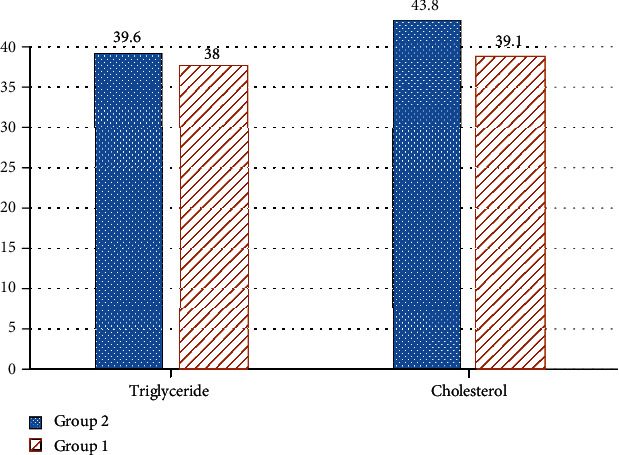
Serum cholesterol and triglyceride levels in both groups.

Further analysis showed a significant decreased serum cholesterol in group 1 of patients (*P* = 0.01), but there were no significant differences between the two groups regarding triglyceride levels (*P* = 0.43). These data are summarized in Table 1 and Figure 1.

Analysis of Pearson correlation indicated significant direct relation between gestational age and both triglyceride and cholesterol levels in neonates without RDS (*r* = 0.633 and 0.668, respectively, *P* < 0.001). On the other hand, there was a significant direct relationship between gestational age and serum triglyceride levels in patients with RDS (*r* = 0.550, *P* < 0.001). No significant relation was detected between gestational age and serum cholesterol levels in neonates with RDS (*P* = 0.95).

Further analysis showed that serum levels of cholesterol were significantly and directly correlated with serum levels of triglyceride in patients without RDS (*r* = 0.569, *P* < 0.001), but no significant relationships were observed in patients with RDS (*P* = 0.16).

We also showed that serum levels of cholesterol in girl patients with RDS were significantly lower than those in the other group (38.8 ± 6.9 vs. 45 ± 10.6, *P* = 0.02), but no significant differences were observed among boys or serum levels of triglyceride in both genders (*P* > 0.05) (Table 2).

## 4. Discussion

Here in the present study, we evaluated and compared the serum levels of triglyceride and cholesterol in neonates with or without RDS. Our data showed a significant decreased serum cholesterol in premature neonates with RDS. But we also showed that there were no significant differences between the two groups regarding triglyceride levels.

Further evaluation indicated a significant direct relation between gestational age and both triglyceride and cholesterol levels in neonates without RDS and a significant direct relationship between gestational age and serum triglyceride levels in patients with RDS. We showed that serum levels of cholesterol in girl patients with RDS were significantly lower than those in boys. The relationship and levels of lipids have been evaluated in premature neonates in previous studies.

In a study by Lane and colleagues in 2002, 39 neonates with RDS and 68 controls without RDS were entered, and the cord serum lipid profiles of patients were evaluated. They showed that the lipid transport across the placenta might be abnormal in patients with RDS. They reported significant lower triglyceride and cholesterol levels in patients with RDS compared to controls [19]. This issue was addressed in a study by Gunes and colleagues in 2007. They showed that total cholesterol, high-density, and low-density lipoprotein (LDL) cholesterol levels were lower in infants with RDS and in their mothers than in controls [20]. These data are in line with the findings of our study. We showed a significant lower cholesterol levels in neonates with RDS. A key point of our study was that we also showed that serum levels of cholesterol in girl patients with RDS were significantly lower than those in boys.

The lower serum cholesterol levels in neonates with RDS were also reported by Yonezawa and others [21]. Wang and others showed that blood lipid levels are related to gestational age and also birth weight. They also suggested that a low triglyceride level might be one of the causes of RDS in preterm infants with a gestational age of 28-30 weeks and a birth weight of ≤1499 g [22]. These data are also in line with our findings. We indicated a significant direct relationship between gestational age and serum triglyceride levels in patients with RDS.

We believe that prematurity and low gestational age could lead to increased chances of RDS by the means of lower lipid levels and also surfactant production. Studies have declared that serum lipid levels could correlate to surfactant production which could indeed reduce the occurrence of RDS [21, 23, 24]. Furthermore, we declared that serum levels of cholesterol in girl patients with RDS were significantly lower than those in boys which has not been reported before.

Different lines of evidence have tried to investigate possible predictors for RDS in premature neonates. Gestational age and lecithin/sphingomyelin ratio, the presence of funisitis, and the help of neonatal lung ultrasound have been investigated in previous studies [25–27]. To the best of our knowledge, no previous study has evaluated cord serum lipid profiles and their use for prediction of RDS in premature neonates. We believe that usage of this system associated with other techniques and factors could be useful in developing a prediction system and, therefore, early interventions in premature neonates who are suspicious of RDS. We also suggest that more studies on larger populations should be performed to clarify the possible prediction value of serum cholesterol for RDS and also its roles in surfactant synthesis.

## 5. Conclusion

Here, we indicated significantly lower cholesterol levels in the cord serum of premature neonates with RDS compared to non-RDS premature neonates. Our data also showed a significant direct relationship between gestational age and serum triglyceride levels in patients with RDS. These data were in line with previous studies. It has been reported that the lower lipid profile could contribute to lower surfactant levels. We also suggested that lower cholesterol levels could be used as a predictor of RDS along with other factors, but more studies on larger populations are still required. One of the limitations of this study was related to the sample size which was small to be generalizable to the whole community. Further studies are needed to investigate the factors that potentially affect the results.

## Figures and Tables

**Table 1:** Serum cholesterol and triglyceride levels.

Variable	Group 1		Group 2		*P* value
	Mean	Standard deviation	Mean	Standard deviation	
Triglyceride	38	8.02	39.6	8.02	0.43
Cholesterol	39.1	6.6	43.8	11	01.0

**Table 2:** Comparison of triglyceride and cholesterol levels based on gender.

Gender		Group 1		Group 2		*P* value
		Mean	Standard deviation	Mean	Standard deviation	
Boy	Triglyceride	37.7	7.5	39.4	11.2	0.52
	Cholesterol	39.3	6.4	42.8	11.4	0.17
Girl	Triglyceride	38.3	8.8	39.8	12.1	0.65
	Cholesterol	38.8	6.9	45	10.6	0.02

## Data Availability

This study is a descriptive-analytical cross-sectional study performed on premature neonates born in Beheshti Hospital of Isfahan University of Medical Sciences in Isfahan in 2018-2019. The study protocol was approved by the ethical committee of Isfahan University of Medical Sciences (IR.MUI.REC.1398.069).
